# (*E*)-4-Hy­droxy-*N*′-(2-hy­droxy-4-meth­oxy­benzyl­idene)benzohydrazide

**DOI:** 10.1107/S1600536812027201

**Published:** 2012-06-30

**Authors:** Hesham Hussein Rassem, Abdussalam Salhin, Baharuddin Bin Salleh, Mohd Mustaqim Rosli, Hoong-Kun Fun

**Affiliations:** a School of Chemical Sciences, Universiti Sains Malaysia, 11800 USM, Penang, Malaysia; b School of Biological Sciences, Universiti Sains Malaysia, 11800 USM, Penang, Malaysia; c X-ray Crystallography Unit, School of Physics, Universiti Sains Malaysia, 11800 USM, Penang, Malaysia

## Abstract

In the title compound, C_15_H_14_N_2_O_4_, the dihedral angle between the benzene rings is 40.59 (4)° and an intra­molecular O—H⋯N hydrogen bond generates an *S*(6) ring. In the crystal, N—H⋯O, O—H⋯O and C—H⋯O inter­actions link the mol­ecules into a three-dimensional network.

## Related literature

For a related sturucture and background to the properties and uses of hydrazones, see: Tameem *et al.* (2008 ▶).
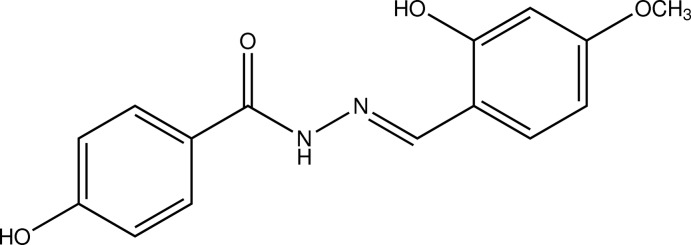


## Experimental

### 

#### Crystal data

C_15_H_14_N_2_O_4_
*M* *_r_* = 286.28
Monoclinic, 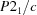

*a* = 15.1982 (2) Å
*b* = 8.2416 (1) Å
*c* = 10.7900 (1) Å
β = 101.173 (1)°
*V* = 1325.91 (3) Å^3^
*Z* = 4
Mo *K*α radiation
μ = 0.11 mm^−1^
*T* = 100 K
0.43 × 0.28 × 0.18 mm

#### Data collection

Bruker SMART APEXII CCD diffractometer
Absorption correction: multi-scan (*SADABS*; Bruker, 2009 ▶) *T*_min_ = 0.956, *T*_max_ = 0.982
21103 measured reflections
5357 independent reflections
4446 reflections with *I* > 2σ(*I*)
*R*_int_ = 0.023

#### Refinement

*R*[*F* ^2^ > 2σ(*F* ^2^)] = 0.043
*wR*(*F* ^2^) = 0.122
*S* = 1.05
5357 reflections
203 parameters
H atoms treated by a mixture of independent and constrained refinement
Δρ_max_ = 0.50 e Å^−3^
Δρ_min_ = −0.26 e Å^−3^

### 

Data collection: *APEX2* (Bruker, 2009 ▶); cell refinement: *SAINT* (Bruker, 2009 ▶); data reduction: *SAINT*; program(s) used to solve structure: *SHELXTL* (Sheldrick, 2008 ▶); program(s) used to refine structure: *SHELXTL*; molecular graphics: *SHELXTL*; software used to prepare material for publication: *SHELXTL* and *PLATON* (Spek, 2009 ▶).

## Supplementary Material

Crystal structure: contains datablock(s) I, global. DOI: 10.1107/S1600536812027201/hb6854sup1.cif

Structure factors: contains datablock(s) I. DOI: 10.1107/S1600536812027201/hb6854Isup2.hkl

Supplementary material file. DOI: 10.1107/S1600536812027201/hb6854Isup3.cml

Additional supplementary materials:  crystallographic information; 3D view; checkCIF report

## Figures and Tables

**Table 1:** Hydrogen-bond geometry (Å, °)

*D*—H⋯*A*	*D*—H	H⋯*A*	*D*⋯*A*	*D*—H⋯*A*
N1—H1*N*1⋯O3^i^	0.859 (15)	2.342 (15)	3.0800 (10)	144.3 (14)
O3—H1*O*3⋯N2	0.866 (18)	1.851 (18)	2.6271 (10)	148.3 (17)
O2—H1*O*2⋯O1^ii^	0.914 (18)	1.766 (18)	2.6713 (9)	170.3 (16)
C5—H5*A*⋯O2^iii^	0.95	2.54	3.2036 (11)	128
C15—H15*A*⋯O1^iv^	0.98	2.53	3.2646 (12)	132

Symmetry codes: (i) 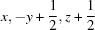; (ii) 
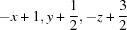
; (iii) 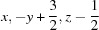; (iv) 
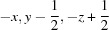
.

**Fig. 1.**
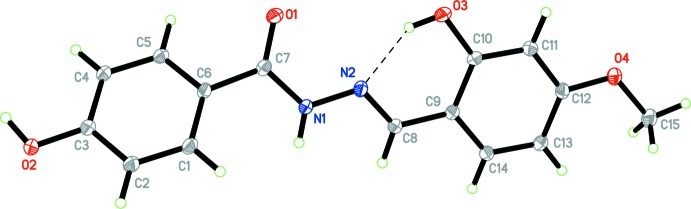
The molecular structure of (I), showing 50% probability displacement ellipsoids. Hydrogen atoms are shown as spheres of arbitrary radius.

**Fig. 2.**
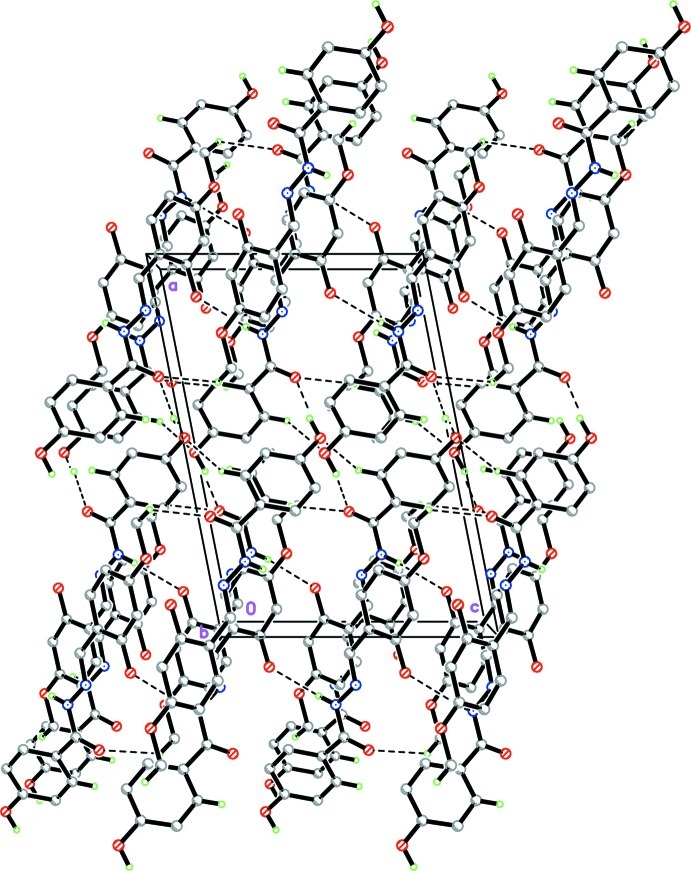
The crystal packing of (I). Dashed lines indicate hydrogen bonds. H atoms not involved in the hydrogen bond interactions have been omitted for clarity.

## supplementary crystallographic information

### Comment

Continuing our interest on the synthesis and application of hydrazone and hydrazide derivatives (Tameem *et al.*, 2008), compound (I) (Fig. 1) was hereby synthesized based on the condensation reaction of 4-hydroxybenzhydrazide and 2-hydroxy-4-methoxybenzaldehyde.

All parameters in (I), are within normal ranges. The dihedral angle between C1—C6 and C9—C14 is 40.59 (4)°. In the molecule, intramolecular interaction of O3—H1O3···N2 form an S(6) hydrogen ring motif. In the crystal structure, the molecules are arranged into a three-dimensional network, connected by N1—H1N1···O3^i^, O2—H1O2···O1^ii^, C5—H5A···O2^iii^ and C15—H15A···O1^iv^ interactions (Table 1).

### Experimental

A solution of 2-hydroxy-4-methoxybenzaldehyde (152 mg, 1 mmol) in methanol (10 ml) was added dropwise to a methanolic solution (10 ml) of 4-hydroxybenzhydrazide (152 mg, 1 mmol) and the mixture was refluxed for 2 h. The resulting solution was condensed on a steam bath to 5 ml and cooled to room temperature. Yellow blocks were separated out, washed with cooled methanol and dried in air.

### Refinement

N and O bound H atoms were located from a difference Fourier map and freely refined. The remaining H atoms were positioned geometrically and refined using a riding model with C—H = 0.95–0.98 Å and *U*_iso_(H) = 1.2*U*_eq_(C) or 1.5*U*_eq_(C) for methyl H atoms. A rotating group model was applied to the methyl group.

### Crystal data

**Table:**

C_15_H_14_N_2_O_4_	*F*(000) = 600
*M**_r_* = 286.28	*D*_x_ = 1.434 Mg m^−^^3^
Monoclinic, *P*2_1_/*c*	Mo *K*α radiation, λ = 0.71073 Å
Hall symbol: -P 2ybc	Cell parameters from 9753 reflections
*a* = 15.1982 (2) Å	θ = 2.7–35.0°
*b* = 8.2416 (1) Å	µ = 0.11 mm^−^^1^
*c* = 10.7900 (1) Å	*T* = 100 K
β = 101.173 (1)°	Block, yellow
*V* = 1325.91 (3) Å^3^	0.43 × 0.28 × 0.18 mm
*Z* = 4	

### Data collection

**Table:**

Bruker SMART APEXII CCD diffractometer	5357 independent reflections
Radiation source: fine-focus sealed tube	4446 reflections with *I* > 2σ(*I*)
Graphite monochromator	*R*_int_ = 0.023
φ and ω scans	θ_max_ = 34.0°, θ_min_ = 2.7°
Absorption correction: multi-scan (*SADABS*; Bruker, 2009)	*h* = −23→22
*T*_min_ = 0.956, *T*_max_ = 0.982	*k* = −10→12
21103 measured reflections	*l* = −16→16

### Refinement

**Table:**

Refinement on *F*^2^	Primary atom site location: structure-invariant direct methods
Least-squares matrix: full	Secondary atom site location: difference Fourier map
*R*[*F*^2^ > 2σ(*F*^2^)] = 0.043	Hydrogen site location: inferred from neighbouring sites
*wR*(*F*^2^) = 0.122	H atoms treated by a mixture of independent and constrained refinement
*S* = 1.05	*w* = 1/[σ^2^(*F*_o_^2^) + (0.0621*P*)^2^ + 0.4059*P*] where *P* = (*F*_o_^2^ + 2*F*_c_^2^)/3
5357 reflections	(Δ/σ)_max_ < 0.001
203 parameters	Δρ_max_ = 0.50 e Å^−^^3^
0 restraints	Δρ_min_ = −0.26 e Å^−^^3^

### Special details

**Table:**

Experimental. The crystal was placed in the cold stream of an Oxford Cryosystems Cobra open-flow nitrogen cryostat (Cosier & Glazer, 1986) operating at 100.0 (1) K.
Geometry. All e.s.d.'s (except the e.s.d. in the dihedral angle between two l.s. planes) are estimated using the full covariance matrix. The cell e.s.d.'s are taken into account individually in the estimation of e.s.d.'s in distances, angles and torsion angles; correlations between e.s.d.'s in cell parameters are only used when they are defined by crystal symmetry. An approximate (isotropic) treatment of cell e.s.d.'s is used for estimating e.s.d.'s involving l.s. planes.
Refinement. Refinement of *F*^2^ against ALL reflections. The weighted *R*-factor *wR* and goodness of fit *S* are based on *F*^2^, conventional *R*-factors *R* are based on *F*, with *F* set to zero for negative *F*^2^. The threshold expression of *F*^2^ > σ(*F*^2^) is used only for calculating *R*-factors(gt) *etc*. and is not relevant to the choice of reflections for refinement. *R*-factors based on *F*^2^ are statistically about twice as large as those based on *F*, and *R*- factors based on ALL data will be even larger.

### Fractional atomic coordinates and isotropic or equivalent isotropic displacement parameters (Å^2^)

**Table:**

	*x*	*y*	*z*	*U*_iso_*/*U*_eq_	
O1	0.31723 (4)	0.31882 (9)	0.55171 (6)	0.01663 (14)	
O2	0.51966 (4)	0.76437 (9)	0.99737 (6)	0.01740 (14)	
O3	0.09841 (4)	0.09665 (9)	0.36222 (6)	0.01737 (14)	
O4	−0.20519 (4)	−0.02273 (9)	0.21628 (6)	0.01959 (14)	
N1	0.19965 (5)	0.38654 (10)	0.64137 (7)	0.01474 (14)	
N2	0.14157 (5)	0.29962 (10)	0.55201 (7)	0.01408 (14)	
C1	0.32956 (6)	0.51526 (12)	0.85518 (8)	0.01642 (16)	
H1A	0.2780	0.4682	0.8784	0.020*	
C2	0.38846 (6)	0.60648 (12)	0.94252 (8)	0.01661 (17)	
H2A	0.3774	0.6206	1.0255	0.020*	
C3	0.46390 (6)	0.67757 (11)	0.90881 (8)	0.01411 (15)	
C4	0.47939 (6)	0.65803 (11)	0.78597 (8)	0.01494 (16)	
H4A	0.5297	0.7085	0.7618	0.018*	
C5	0.42107 (6)	0.56481 (11)	0.69974 (8)	0.01445 (15)	
H5A	0.4324	0.5500	0.6170	0.017*	
C6	0.34575 (5)	0.49233 (11)	0.73292 (8)	0.01322 (15)	
C7	0.28800 (5)	0.39174 (11)	0.63549 (8)	0.01329 (15)	
C8	0.05729 (6)	0.31976 (11)	0.55158 (8)	0.01372 (15)	
H8A	0.0393	0.3916	0.6109	0.016*	
C9	−0.01035 (5)	0.23455 (10)	0.46203 (8)	0.01278 (15)	
C10	0.01179 (6)	0.12335 (11)	0.37274 (8)	0.01310 (15)	
C11	−0.05540 (6)	0.03854 (11)	0.29367 (8)	0.01457 (15)	
H11A	−0.0402	−0.0385	0.2357	0.017*	
C12	−0.14544 (6)	0.06598 (11)	0.29894 (8)	0.01454 (15)	
C13	−0.16902 (6)	0.17757 (11)	0.38464 (8)	0.01555 (16)	
H13A	−0.2302	0.1976	0.3872	0.019*	
C14	−0.10109 (6)	0.25820 (11)	0.46569 (8)	0.01491 (16)	
H14A	−0.1166	0.3319	0.5257	0.018*	
C15	−0.29779 (6)	−0.01043 (14)	0.22528 (9)	0.02144 (19)	
H15A	−0.3334	−0.0854	0.1651	0.032*	
H15B	−0.3187	0.1008	0.2058	0.032*	
H15C	−0.3046	−0.0383	0.3112	0.032*	
H1N1	0.1785 (10)	0.4363 (19)	0.6991 (14)	0.029 (4)*	
H1O3	0.1324 (12)	0.155 (2)	0.4189 (18)	0.047 (5)*	
H1O2	0.5723 (12)	0.788 (2)	0.9722 (16)	0.039 (4)*	

### Atomic displacement parameters (Å^2^)

**Table:**

	*U*^11^	*U*^22^	*U*^33^	*U*^12^	*U*^13^	*U*^23^
O1	0.0124 (3)	0.0221 (3)	0.0158 (3)	0.0004 (2)	0.0038 (2)	−0.0021 (2)
O2	0.0132 (3)	0.0228 (3)	0.0166 (3)	−0.0040 (2)	0.0039 (2)	−0.0049 (2)
O3	0.0116 (3)	0.0226 (3)	0.0186 (3)	0.0009 (2)	0.0045 (2)	−0.0035 (2)
O4	0.0142 (3)	0.0253 (4)	0.0187 (3)	−0.0046 (2)	0.0017 (2)	−0.0060 (3)
N1	0.0103 (3)	0.0188 (4)	0.0152 (3)	−0.0015 (2)	0.0027 (2)	−0.0035 (3)
N2	0.0116 (3)	0.0161 (3)	0.0141 (3)	−0.0019 (2)	0.0016 (2)	−0.0008 (2)
C1	0.0126 (3)	0.0224 (4)	0.0149 (3)	−0.0037 (3)	0.0043 (3)	0.0006 (3)
C2	0.0143 (4)	0.0223 (4)	0.0139 (3)	−0.0030 (3)	0.0044 (3)	−0.0002 (3)
C3	0.0113 (3)	0.0161 (4)	0.0149 (3)	−0.0001 (3)	0.0025 (3)	−0.0004 (3)
C4	0.0121 (3)	0.0178 (4)	0.0157 (3)	−0.0022 (3)	0.0046 (3)	−0.0008 (3)
C5	0.0125 (3)	0.0176 (4)	0.0140 (3)	−0.0012 (3)	0.0043 (3)	−0.0002 (3)
C6	0.0105 (3)	0.0155 (4)	0.0135 (3)	−0.0010 (3)	0.0018 (2)	0.0008 (3)
C7	0.0115 (3)	0.0150 (4)	0.0132 (3)	−0.0004 (3)	0.0022 (2)	0.0022 (3)
C8	0.0119 (3)	0.0153 (4)	0.0139 (3)	−0.0003 (3)	0.0025 (2)	−0.0010 (3)
C9	0.0112 (3)	0.0136 (4)	0.0134 (3)	−0.0002 (3)	0.0022 (2)	0.0001 (3)
C10	0.0117 (3)	0.0151 (4)	0.0130 (3)	0.0009 (3)	0.0036 (2)	0.0011 (3)
C11	0.0151 (4)	0.0152 (4)	0.0138 (3)	−0.0010 (3)	0.0035 (3)	−0.0010 (3)
C12	0.0136 (3)	0.0160 (4)	0.0135 (3)	−0.0025 (3)	0.0015 (3)	0.0005 (3)
C13	0.0112 (3)	0.0179 (4)	0.0176 (4)	−0.0004 (3)	0.0028 (3)	−0.0017 (3)
C14	0.0122 (3)	0.0158 (4)	0.0168 (4)	0.0002 (3)	0.0030 (3)	−0.0026 (3)
C15	0.0145 (4)	0.0278 (5)	0.0210 (4)	−0.0049 (3)	0.0008 (3)	−0.0033 (4)

### Geometric parameters (Å, º)

**Table:**

O1—C7	1.2375 (11)	C4—H4A	0.9500
O2—C3	1.3525 (10)	C5—C6	1.3979 (12)
O2—H1O2	0.915 (18)	C5—H5A	0.9500
O3—C10	1.3610 (10)	C6—C7	1.4849 (12)
O3—H1O3	0.866 (19)	C8—C9	1.4487 (11)
O4—C12	1.3568 (10)	C8—H8A	0.9500
O4—C15	1.4329 (12)	C9—C14	1.4009 (12)
N1—C7	1.3571 (11)	C9—C10	1.4166 (12)
N1—N2	1.3756 (10)	C10—C11	1.3862 (12)
N1—H1N1	0.858 (16)	C11—C12	1.3990 (12)
N2—C8	1.2907 (11)	C11—H11A	0.9500
C1—C2	1.3885 (12)	C12—C13	1.3992 (13)
C1—C6	1.4008 (12)	C13—C14	1.3865 (12)
C1—H1A	0.9500	C13—H13A	0.9500
C2—C3	1.3971 (12)	C14—H14A	0.9500
C2—H2A	0.9500	C15—H15A	0.9800
C3—C4	1.3997 (12)	C15—H15B	0.9800
C4—C5	1.3864 (12)	C15—H15C	0.9800
			
C3—O2—H1O2	111.6 (11)	N2—C8—C9	121.03 (8)
C10—O3—H1O3	107.8 (12)	N2—C8—H8A	119.5
C12—O4—C15	117.28 (7)	C9—C8—H8A	119.5
C7—N1—N2	119.35 (7)	C14—C9—C10	118.30 (8)
C7—N1—H1N1	122.1 (10)	C14—C9—C8	119.27 (8)
N2—N1—H1N1	118.5 (10)	C10—C9—C8	122.40 (8)
C8—N2—N1	115.92 (7)	O3—C10—C11	118.44 (8)
C2—C1—C6	120.29 (8)	O3—C10—C9	121.49 (8)
C2—C1—H1A	119.9	C11—C10—C9	120.07 (8)
C6—C1—H1A	119.9	C10—C11—C12	120.21 (8)
C1—C2—C3	120.27 (8)	C10—C11—H11A	119.9
C1—C2—H2A	119.9	C12—C11—H11A	119.9
C3—C2—H2A	119.9	O4—C12—C11	114.94 (8)
O2—C3—C2	118.12 (8)	O4—C12—C13	124.33 (8)
O2—C3—C4	122.18 (8)	C11—C12—C13	120.73 (8)
C2—C3—C4	119.70 (8)	C14—C13—C12	118.49 (8)
C5—C4—C3	119.76 (8)	C14—C13—H13A	120.8
C5—C4—H4A	120.1	C12—C13—H13A	120.8
C3—C4—H4A	120.1	C13—C14—C9	122.16 (8)
C4—C5—C6	120.91 (8)	C13—C14—H14A	118.9
C4—C5—H5A	119.5	C9—C14—H14A	118.9
C6—C5—H5A	119.5	O4—C15—H15A	109.5
C5—C6—C1	119.05 (8)	O4—C15—H15B	109.5
C5—C6—C7	117.28 (7)	H15A—C15—H15B	109.5
C1—C6—C7	123.67 (8)	O4—C15—H15C	109.5
O1—C7—N1	121.15 (8)	H15A—C15—H15C	109.5
O1—C7—C6	122.80 (8)	H15B—C15—H15C	109.5
N1—C7—C6	116.04 (7)		
			
C7—N1—N2—C8	−169.85 (8)	N2—C8—C9—C14	179.21 (8)
C6—C1—C2—C3	0.66 (14)	N2—C8—C9—C10	1.28 (13)
C1—C2—C3—O2	−179.55 (8)	C14—C9—C10—O3	178.72 (8)
C1—C2—C3—C4	0.73 (14)	C8—C9—C10—O3	−3.34 (13)
O2—C3—C4—C5	178.60 (8)	C14—C9—C10—C11	−1.39 (13)
C2—C3—C4—C5	−1.68 (14)	C8—C9—C10—C11	176.55 (8)
C3—C4—C5—C6	1.27 (13)	O3—C10—C11—C12	−178.13 (8)
C4—C5—C6—C1	0.11 (13)	C9—C10—C11—C12	1.98 (13)
C4—C5—C6—C7	−178.74 (8)	C15—O4—C12—C11	174.52 (8)
C2—C1—C6—C5	−1.08 (14)	C15—O4—C12—C13	−5.41 (13)
C2—C1—C6—C7	177.70 (9)	C10—C11—C12—O4	179.35 (8)
N2—N1—C7—O1	−0.48 (13)	C10—C11—C12—C13	−0.72 (13)
N2—N1—C7—C6	178.13 (7)	O4—C12—C13—C14	178.82 (8)
C5—C6—C7—O1	28.68 (13)	C11—C12—C13—C14	−1.10 (13)
C1—C6—C7—O1	−150.12 (9)	C12—C13—C14—C9	1.70 (14)
C5—C6—C7—N1	−149.91 (8)	C10—C9—C14—C13	−0.47 (13)
C1—C6—C7—N1	31.29 (12)	C8—C9—C14—C13	−178.48 (8)
N1—N2—C8—C9	−179.89 (8)		

### Hydrogen-bond geometry (Å, º)

**Table:**

*D*—H···*A*	*D*—H	H···*A*	*D*···*A*	*D*—H···*A*
N1—H1*N*1···O3^i^	0.859 (15)	2.342 (15)	3.0800 (10)	144.3 (14)
O3—H1*O*3···N2	0.866 (18)	1.851 (18)	2.6271 (10)	148.3 (17)
O2—H1*O*2···O1^ii^	0.914 (18)	1.766 (18)	2.6713 (9)	170.3 (16)
C5—H5*A*···O2^iii^	0.95	2.54	3.2036 (11)	128
C15—H15*A*···O1^iv^	0.98	2.53	3.2646 (12)	132

Symmetry codes: (i) *x*, −*y*+1/2, *z*+1/2; (ii) −*x*+1, *y*+1/2, −*z*+3/2; (iii) *x*, −*y*+3/2, *z*−1/2; (iv) −*x*, *y*−1/2, −*z*+1/2.
